# Young Breast Cancer: Novel Gene Methylation in WBC

**DOI:** 10.31557/APJCP.2021.22.8.2371

**Published:** 2021-08

**Authors:** Mehrdad Noruzinia, Javad Tavakkoly-Bazzaz, Mohammad Ahmadvand, Fatemeh Azimi, Ali Dehghanifard, Golnaz Khakpour

**Affiliations:** 1 *Department of Medical Genetics, Faculty of Medical Sciences, Tarbiat Modares University, Tehran, Iran. *; 2 *Department of Medical Genetics, School of Medicine, Tehran University of Medical Sciences, Tehran, Iran. *; 3 *Hematology, Oncology, and Stem Cell Transplantation Research Center, Shariati Hospital, Tehran University of Medical Sciences, Tehran, Iran. *; 4 *Eye Research Center, the Five Senses Institute, Rassoul Akram Hospital, Iran University of Medical Sciences, Tehran, Iran. *; 5 *Department of Vaccination, Pasteur Institute of Iran, Tehran, Iran. *

**Keywords:** ZNF154, BCL9, HOXD9 methylation

## Abstract

**Introduction:**

Breast cancer is a highly diverse disease, and epigenomic alterations, as principle changes in the pathogenesis of breast cancer, have recently been noticed in epimarker research on peripheral blood.

**Methods:**

In this study, DNA samples isolated from the white blood cells of 30 breast cancer patients were compared to 30 healthy controls using methylated DNA immunoprecipitation microarray (MeDIP-chip) to determine differentially methylated region as a potential epimarker in cancer and control cases.

**Results:**

A total of 1799 differentially methylated regions were identified, including ZNF154, BCL9, and HOXD9, in which significant methylation differences were confirmed in breast cancer patients through a quantitative real-time polymerase chain reaction. Differential methylation of the mentioned genes has been reported in different cancer tissues and cell-free DNA, including breast cancer. Methylation of those genes listed in the white blood cells of our young patients not only relates to their importance in the pathogenesis of breast cancer but may also highlight their potential as primary epimarkers that can warrant further evaluation in large cohort studies. It is important to note that methylation alteration in WBC, as well as genetic mutation, can be identified years before cancer development, which emphasizes this issue as a potential screening marker.

## Introduction

Breast cancer is a very heterogeneous disease with multiple histological and molecular subtypes that have various clinical behaviors and therapeutic responses. Since breast cancer remains a leading cause of cancer-related deaths, early diagnosis may lead to improved treatment and better outcomes.

Epimutation (fault in epigenetic manipulation) and genetic factors are powerful mechanisms in breast cancer development. Epigenetics as a heritable alteration without any changes in the DNA sequence can lead to the initiation and progression of disease by dysregulation of the gene expression, and methylation changes in promoter and intragenic regions have different effects on gene expression. Regardless of their influences on gene expression, these alterations have been identified in different cancers. 

The various types of methylation changes that occur in different individuals can modify their genetic susceptibility to breast cancer. Moreover, it seems that epigenetic marks in the normal tissues could be a predictor of cancer development in the future. So, methylation changes could be considered as an early screening method in breast cancer patients. Since biopsy of the targeted tissue as well as breast is not always reasonable for screening, peripheral blood can be used as a surrogate source for methylation analysis. 

However, it is not clear if the methylation of some genes in WBC is a sign of methylation in the primary tissue or if it is merely a trace of external exposures. Recent studies identified that methylation alterations could be a predictor of some cancers such as bladder, ovarian, pancreas, and breast. It is important to note that some methylation changes in WBC are due to aging, and there could be a link between cancer and aging. In this respect, we removed the aging effect and aimed to evaluate WBC methylation in young breast cancer patients compared to a normal control group to seek a potential epimarker using the whole genome approach called the methylated DNA immunoprecipitation microarray (MeDIP-chip).

## Metherials and Methods

*Cases and Controls*

Our study population consisted of 30 breast cancer patients recruited from Imam Khomeini Hospital. These patients had no family history of cancer and had undergone breast surgery but had not yet any chemotherapy. The control group consisted of 30 unrelated healthy volunteers without any medical history of cancer or other chronic diseases. The control group underwent sonography or mammography for breast cancer screening. 

Both the cancer cases and the control group were between 25-35 years of age (mean age in both groups: 30 ± 0.40) and were informed and gave consent by signing consent forms according to the Ethical Committee of Tehran University of Medical Sciences. All patients and controls were of the same ethnicity (background). Blood samples were collected before any intervention, including surgery, chemotherapy, or radiotherapy.

*DNA isolation and DNA methylation analysis*

Genomic DNA was extracted from whole blood using the High Pure PCR Preparation kit (Roch). The isolated DNA was stored at –20 °C until the next procedure. 

Subsequently, it was sonicated to obtain the size range of an optimal fragment (200–1000 bp) for MeDIP analysis. In the MeDIP process, all methylated DNA sequences were pulled down by a monoclonal antibody against 5-methylcytosine, using the Methylated DNA Capture Kit (Epigentek) according to the manufacturer’s instruction. To evaluate the MeDIP procedure, isolated methylated DNA (from the MeDIP procedure) and input DNA samples (that were the fractions of sonicated DNA), including both methylated and unmethylated sequences (as a references DNA), were compared in the quantitative Real-time polymerase chain reaction (qPCR) using primers specific to *H19* gene as the endogenous positive control (*hemimethylated* gene) and GAPDH as the *unmethylated* gene.

Enrichment = (E target) ∆ Cq target (Input-MeDIP) / (E reference) ∆ Cq reference (Input-MeDIP) (Khakpour et al., 2017)

Where “E” is the efficiency of amplification, “target” is the methylated region of interest, and “reference” is the normalizer (a DNA region that is known, or expected, to be unmethylated in all the samples). The enrichment ranges of 25–27 were included for the following steps of analysis. Because the concentrations of immunoprecipated (IP) samples are too little for analysis in microarrays, the IP and input fractions obtained from all cases and controls were amplified by the GenomePlex Complete Genome (WGA) Amplified Kit (WGA). (# WGA2; Sigma) -Aldrich.

The input and IP DNA fractions were labeled with Cy3 and Cy5 dyes, respectively.They were then hybridized to the NimbleGen Human DNA Methylation 3×720K Promoter plus CpG Island Array. It is a multiplex slide with three identical arrays per slide, in which each array contains 27,728 CpG islands annotated by UCSC and 22,532 well-characterized RefSeq promoter regions (from about −2440 to +610 bp of the transcription start sites (TSSs) that are completely covered by ~720,000 probes.

*Statistical analysis*

The scan was performed with an Axon GenePix 4000B microarray scanner. The raw data created by NimbleScan software were normalized using, quantile normalization, median-centering, and linear smoothing by Bioconductor packages limma, Ringo, and MEDME. After normalization, log2 normal ratio data was generated for each sample. Enriched peaks should be generated from normalized adjacent probes rised significantly above a set threshold; the area is assigned to an enrichment peak (EP). Nimble Scan detected peaks by searching at least two probes above log2-ratio data. Each probe obtained a p-value of −log10 from the Kolmogorov-Smirnov (KS) window test. If several the minimum cut-off value of P-value (−log10) 2.

The average of the log2-ratio value (Experiment and Control) was used to calculate the M′ value to compare the probe-specific differentially enriched regions between the two studied groups. The Nimble Scan sliding-window peak-finding algorithm was then re-run on those data to find the differential enrichment peaks (DEPs). The T-test was used for microarray data of replication samples to determine the difference in each probe used in microarray between different groups. Probes with a p value < 0.05 were considered as differentially methylated probes. 

These differentially methylated regions (DMRs) were mostly annotated as island CpG or promoter according to their position in the genome. Finally, some candidates’ genes were selected from DMRs found in the CpG promoters and islands to be confirmed by Real-time PCR.

*Real-time PCR*

Real-time PCR was used to verify the selected DMR identified by array results. The qPCR program was initiated with denaturation at 95°C for 15 min followed by 40 cycles of 95°C for 30 s, 59°C–61°C for 30 s, 72°C for 30 s, and a final extension at 72°C for 10 min. The Real-time PCR reaction was comprised of Maxima™ SYBR Green qPCR Master Mix 2× (Fermentas), 5 ng of DNA, and 10 pmol of specific primer pairs adjusted with nuclease-free ddH2O up to a final volume of 10 µL.

The difference of methylation between the cancer cases and controls was determined using the 2^−ΔΔCT^ method with H19 as the normalizer and reference gene for every specific locus. The results were tested by doing real-time PCR on the serial dilutions of DNA within the range of 10-fold change as a template using the specific primer pairs of each gene and the *H19* gene. The qPCR was performed in triplicate for each gene on all samples, and the mean was then utilized in the relative quantification (RQ) method.

## Results

We identified 1799 DMRs in the promoter and other genomic regions, including intragenic and intergenic, which were differentially methylated between the patients and the normal samples. 

Generally, 39.1% of the DMRs were methylated in the cancer patients while the remaining (60.9%) DMRs were methylated in the controls, which were then remarked as hypermethylated and hypomethylated regions, respectively, based on the statistical evaluation of raw data.

To assess the potential of DMR-associated genes as epimarkers of breast cancer, a locus-specific Real-time PCR was carried out on regions with highly differentiated methylation between the cancer cases and the controls.

In the present study, *5 DMRs, including HOXD9, ZNF154, BCL9, TDRD10*, and *ANKRD53* genes, were selected to be candidates for further validation (based on differentially methylated probes) with Real-time PCR (3 of them, including promoter regions, had significant methylation differences in the patients compared to the healthy controls (t-test; p < 0.05):

1. HOXD9: chr2: 176689940–176690795 HG18), which was hypomethylated in the promoter region.

2. ZNF154: chr19: 62912391- 62903621 HG18), which was hypermethylated in the promoter region.

3. BCL9: chr1: 145479805- 145564639 HG18), which was hypomethylated in the promoter region.

*Real-time PCR validation*

Real-time PCR was performed to check the methylation difference of *HOXD9, ZNF154, BCL9, TDRD10*, and *ANKRD53* genes between the cancer cases and controls. Calculated based on the Ct variation from the Ct mean value, the CV value of *HOXD9, ZNF154, BCL9* genes in the case group was low (<5%). Also, the mean value comparison in the mentioned genes was significant between the cancer cases and controls (p < 0.05). It seems that the amount of RQ in the mentioned genes is significant between the cancer cases and control groups (Table 1).

In summary, the results showed that the methylation pattern of *HOXD9, ZNF154, *and* BCL9* genes in peripheral blood leukocytes could be used as a potential biomarker for breast cancer risk assessment.

**Table 1:** The Mean of ΔCT and CV Values between the Cancer Cases and Control Samples

Gene	Breast cancer cases		Healthy controls		p*
	Mean ΔCT (n = 30)	CV (%)	Mean ΔCT (n = 30)	CV (%)	
*HOXD9*	21.03	0.05	23.04	0.07	0.027
*ZNF154*	23.05	1.74	24.6	1.03	0.025
*BCL9*	22.01	0.09	20.08	1.05	0.021
*TDRD10*	23.04	7.22	24.11	0.78	0.081
*ANKRD53*	21.08	8.80	23.04	1.02	0.076

## Discussion

DNA methylation is an important epigenetic alteration that can change gene expression and is usually disrupted in cancer. In young breast cancer patients without a family history or genetic mutation, identifying epigenetic alteration in their peripheral blood may pave the way for early screening, diagnosis, and targeted treatment. In this study, 30 breast cancer patients were compared with 30 healthy controls to determine if their peripheral blood methylation is a potential epimarker. This study followed our previous experiment that suggested four potential epimarker in WBC of young breast cancer patients. 

Three novel DMRs located in promoter regions of *HOXD9, ZNF154*, and* BCL9* genes were confirmed in the peripheral blood of breast cancer patients. The *HOXD9* gene belongs to the homeobox family. This gene is one of several homeobox *HOXD* genes located on the 2q31-2q37 chromosome regions. The CpG island localized in the promoter of this gene was shown to be significantly hypomethylated in cancer patients compared to healthy controls. *HOXD9s* were previously enlisted as genes that are methylated in the serum cell-free DNA of Cholangiocarcinoma patients as compared to other biliary diseases.

Kondo et al., (2018) reported HOXD9 methylation in thymomas was significantly higher than in the normal thymus, and epigenetic alteration may be relevant to the progression of malignancy in thymic epithelial tumors. Although there is an inconsistency between two recent studies and our data, the overexpression of HOXD9 in different cancers agrees with the hypomethylation of this gene in our findings. In addition to our findings, because of hypomethylation and overexpression of HOXD9 in young breast cancer tissues. we speculate that WBC methylation could be a substitute for tissue epigenetic alteration in young breast cancer research.

The second DMR was ZNF154, which is a member of the KRAB-ZNF family of transcriptional regulators. Their members are thought to act in normal and abnormal cell growth and differentiation. *ZNF154* genes are located on the 19q13.43 chromosome region. The CpG island localized in the promoter of this gene was shown to be significantly hypermethylated in cancer patients compared to healthy controls. It has been proven that the hypermethylation and low expression of ZNF154 in different solid tumors, including prostate cancer and nasopharyngeal carcinoma, could be a predictive marker. Indeed, the hypermethylation of ZNF154 in the tissue samples and plasma collected in the early stages of different solid tumors, including breast cancer is consistent with our data. It is important to note that cell-free DNA originates from tumor tissue, and methylation in WBC is different from cell-free DNA. Accordingly, in epimarker research, the selection of WBC or cell-free DNA as a surrogate marker of methylation alteration in tumor tissue is a challenging issue.

It is worth noting that methylation alteration in WBC may be detectable years before cancer development and could be considered a potent epigenetic blood-based biomarker for screening, which is not possible with cell-free DNA. The third differentially methylated region was BCL9; its promoter was found to be significantly hypermethylated in cancer patients compared to healthy controls. B-cell lymphoma 9 (BCL9) oncogene functions as a transcriptional co-activator of the Wnt/β-catenin pathway. 

A recent publication reported that BCL9 is a breast cancer-related gene that contributes to invasion and EMT in breast ductal carcinoma. Another study demonstrated that BCL9 methylation status and its expression pattern are definitely related to ERBB2 and HER2 function in breast cancers ERBB2 and HER2 are both biomarkers of molecular subtypes of breast cancer; this functional link could consider BCL9 as a potential biomarker at the methylation level. It is noteworthy that targeting of BCL9 inhibits DCIS in both the cell line and animal model moreover, our data may emphasize the role of BCL9 in breast cancer development.

It should also be mentioned that detecting methylation in *CpG-rich* genes conserved against methylation, including those above, is an encouraging subject that could be considered in future epimarker research. There have been many studies done on methylation of breast tissue and a few studies on methylation of WBC in peripheral blood of breast cancer patients. However, the relationship between methylation patterns in WBC and breast tissue remains a critical question in biomarker studies.

There are conflicting data concerning the correlation between the methylation pattern of breast cancer tissue and methylation alteration in WBC, which we categorized in a previous study (Khakpour et al., 2015). Methylation of those genes listed in the white blood cells of our young patients not only relates to their importance in the pathogenesis of breast cancer but may also highlight their potential as primary epimarkers. This potential warrants further evaluation in large cohort studies. Taken together, the epigenetic status of WBC may be a surrogate for environmental exposure and genetic variability. However, the alteration of the methylation signature of specific genes may expose new targets of the first hit at the methylation level that susceptible young women with no genetic mutation. Further studies are necessary to confirm the methylation status of the mentioned DMRs in tissues and WBCs of breast cancer patients in large cohort studies and different populations with racial disparities.

## Author Contribution Statement

M.N. and J.T designed the study; M.A. and A.D. did the technical issues. F.A. drafted the manuscript and revised the manuscript; G.K. supervised the study.

## References

[B1] Bacos K, Gillberg L, Volkov P (2016). Blood-based biomarkers of age-associated epigenetic changes in human islets associate with insulin secretion and diabetes. Nat Commun.

[B2] Bastien RR, Rodríguez-Lescure Á, Ebbert MT (2012). PAM50 breast cancer subtyping by RT-qPCR and concordance with standard clinical molecular markers. BMC Med Genomics.

[B3] Bhatlekar S, Fields JZ, Boman BM (2018). Role of HOX genes in stem cell differentiation and cancer. Stem Cells Int.

[B4] Cash HL, Tao L, Yuan JM (2012). LINE-1 hypomethylation is associated with bladder cancer risk among nonsmoking Chinese. Int J Cancer.

[B5] Cho YH, McCullough LE, Gammon MD (2015). Promoter hypermethylation in white blood cell DNA and breast cancer risk. J Cancer.

[B6] Del Campo M, Jones MC, Veraksa AN (1999). Monodactylous limbs and abnormal genitalia are associated with hemizygosity for the human 2q31 region that includes the HOXD cluster. Am J Hum Genet.

[B7] Elsarraj HS, Hong Y, Limback D (2020). BCL9/STAT3 regulation of transcriptional enhancer networks promote DCIS progression. NPJ Breast Cancer.

[B8] Elsarraj HS, Hong Y, Valdez KE (2015). Expression profiling of in vivo ductal carcinoma in situ progression models identified B cell lymphoma-9 as a molecular driver of breast cancer invasion. Breast Cancer Res.

[B9] Flanagan JM, Munoz-Alegre M, Henderson S (2009). Gene-body hypermethylation of ATM in peripheral blood DNA of bilateral breast cancer patients. Hum Mol Genet.

[B10] Heyn H, Carmona FJ, Gomez A (2013). DNA methylation profiling in breast cancer discordant identical twins identifies DOK7 as novel epigenetic biomarker. Carcinogenesis.

[B11] Hu Y, Qi M-F, Xu Q-L (2017). Candidate tumor suppressor ZNF154 suppresses invasion and metastasis in NPC by inhibiting the EMT via Wnt/β-catenin signalling. Oncotarget.

[B12] Iwamoto T, Yamamoto N, Taguchi T (2011). BRCA1 promoter methylation in peripheral blood cells is associated with increased risk of breast cancer with BRCA1 promoter methylation. Breast Cancer Res Treat.

[B13] Khakpour G, Noruzinia M, Izadi P (2017). Methylomics of breast cancer: seeking epimarkers in peripheral blood of young subjects. Tumor Biol.

[B14] Khakpour G, Pooladi A, Izadi P (2015). DNA methylation as a promising landscape: A simple blood test for breast cancer prediction. Tumor Biol.

[B15] Kondo K, Kishibuchi R, Soejima S (2018). P1 14-16 DNA methylation of GNG4 GHSR, HOXD9 and SALL3 genes predict malignant behavior of thymic epithelial tumors. J Thorac Oncol.

[B16] Li H, Huang C-J, Choo K-B (2002). Expression of homeobox genes in cervical cancer. Gynecol Oncol.

[B17] Li L, Choi J-Y, Lee K-M (2012). DNA methylation in peripheral blood: a potential biomarker for cancer molecular epidemiology. J Epidemiol.

[B18] Lo P-K, Sukumar S (2008). Epigenomics and breast cancer. Pharmacogenomics.

[B19] Lønning PE, Berge EO, Bjørnslett M (2018). White blood cell BRCA1 promoter methylation status and ovarian cancer risk. Ann Intern Med.

[B20] Margolin G, Petrykowska HM, Jameel N (2016). Robust detection of DNA hypermethylation of ZNF154 as a pan-cancer locus with in silico modeling for blood-based diagnostic development. J Mol Diagn.

[B22] Moore LE, Pfeiffer RM, Poscablo C (2008). Genomic DNA hypomethylation as a biomarker for bladder cancer susceptibility in the Spanish Bladder Cancer Study: a case–control study. Lancet Oncol.

[B23] Neale RE, Clark PJ, Fawcett J (2014). Association between hypermethylation of DNA repetitive elements in white blood cell DNA and pancreatic cancer. Cancer Epidemiol.

[B24] Oltra SS, Peña-Chilet M, Flower K (2019). Acceleration in the DNA methylation age in breast cancer tumours from very young women. Sci Rep.

[B25] Pedersen KS, Bamlet WR, Oberg AL (2011). Leukocyte DNA methylation signature differentiates pancreatic cancer patients from healthy controls. PLoS One.

[B26] Riley PA (2018). Epimutation and cancer: Carcinogenesis viewed as error-prone inheritance of epigenetic information. J Oncol.

[B27] Sánchez-Vega F, Gotea V, Petrykowska HM (2013). Recurrent patterns of DNA methylation in the ZNF154, CASP8, and VHL promoters across a wide spectrum of human solid epithelial tumors and cancer cell lines. Epigenetics.

[B28] Takada K, Zhu D, Bird GH (2012). Targeted disruption of the BCL9/β-catenin complex inhibits oncogenic Wnt signaling. Sci Transl Med.

[B29] Teschendorff AE, Menon U, Gentry-Maharaj A (2009). An epigenetic signature in peripheral blood predicts active ovarian cancer. PloS One.

[B30] Toya H, Oyama T, Ohwada S (2007). Immunohistochemical expression of the β-catenin-interacting protein B9L is associated with histological high nuclear grade and immunohistochemical ErbB2/HER-2 expression in breast cancers. Cancer Sci.

[B31] Verma M, Rogers S, Divi RL (2014). Epigenetic research in cancer epidemiology: trends, opportunities, and challenges. Cancer Epidemiol Biomarkers Prev.

[B32] Wasenang W, Chaiyarit P, Proungvitaya S (2019). Serum cell-free DNA methylation of OPCML and HOXD9 as a biomarker that may aid in differential diagnosis between cholangiocarcinoma and other biliary diseases. Clin Epigenetics.

[B33] Wilhelm CS, Kelsey KT, Butler R (2010). Implications of LINE1 methylation for bladder cancer risk in women. Clin Cancer Res.

[B34] Yersal O, Barutca S (2014). Biological subtypes of breast cancer: Prognostic and therapeutic implications. World J Clin Oncol.

[B35] Zhang W, Shu P, Wang S (2018). ZNF154 is a promising diagnosis biomarker and predicts biochemical recurrence in prostate cancer. Gene.

[B36] Zhu H, Dai W, Li J (2019). HOXD9 promotes the growth, invasion and metastasis of gastric cancer cells by transcriptional activation of RUFY3. J Exp Clin Cancer Res.

